# Dosimetric Comparison and Potential for Improved Clinical Outcomes of Paediatric CNS Patients Treated with Protons or IMRT

**DOI:** 10.3390/cancers7020706

**Published:** 2015-04-28

**Authors:** Kris S. Armoogum, Nicola Thorp

**Affiliations:** 1 Department of Radiotherapy Physics, Royal Derby Hospital, Derby Hospitals NHS Foundation Trust, Uttoxeter Road, Derby DE22 3NE, UK; 2 The Clatterbridge Cancer Centre NHS Foundation Trust, Clatterbridge Road, Bebington, Wirral CH63 4JY, UK; E-Mail: nicky.thorp@clatterbridgecc.nhs.uk

**Keywords:** proton therapy, IMRT, paediatric, CNS, medulloblastoma, cranio-pharyngioma, low grade glioma, ependymoma

## Abstract

*Background*: We compare clinical outcomes of paediatric patients with CNS tumours treated with protons or IMRT. CNS tumours form the second most common group of cancers in children. Radiotherapy plays a major role in the treatment of many of these patients but also contributes to late side effects in long term survivors. Radiation dose inevitably deposited in healthy tissues outside the clinical target has been linked to detrimental late effects such as neurocognitive, behavioural and vascular effects in addition to endocrine abnormalities and second tumours. *Methods*: A literature search was performed using keywords: protons, IMRT, CNS and paediatric. Of 189 papers retrieved, 10 were deemed relevant based on title and abstract screening. All papers directly compared outcomes from protons with photons, five papers included medulloblastoma, four papers each included craniopharyngioma and low grade gliomas and three papers included ependymoma. *Results*: This review found that while proton beam therapy offered similar clinical target coverage, there was a demonstrable reduction in integral dose to normal structures. *Conclusions*: This in turn suggests the potential for superior long term outcomes for paediatric patients with CNS tumours both in terms of radiogenic second cancers and out-of-field adverse effects.

## 1. Introduction

Central Nervous System (CNS) tumours are the second most common cancers in children, accounting for approximately 25% of all childhood cancers. The highest incidence rates for all childhood cancers combined are in the under-fives for both sexes, with almost half of all cases being diagnosed in this age group. Improvements in therapy especially over the last decade has improved life expectancy in paediatric cancer patients, with a 5-year overall survival (OS) currently exceeding 80% [1]. Radiation therapy used in combination with surgery and chemotherapy is very effective in the management of paediatric CNS tumours, but is accompanied by the risk of late toxicity and secondary tumor induction [2]. Regardless of the delivery modality, radiation dose inevitably deposited in healthy tissues outside the clinical target has been linked to detrimental late effects such as neurocognitive, behavioural and vascular effects in addition to endocrine abnormalities and second tumours. These late effects are compounded by the increasing long-term survival rates, and survivors of childhood CNS cancers face the prospect of developing secondary cancers several decades after primary treatment. Radiation treatment approaches have sought to develop strategies to reduce exposure to adjacent healthy tissues. They include intensity modulated photon radiotherapy (IMRT) and more recently, proton beam therapy (PBT) [3].

Proton radiation consists of charged particles with mass, which deposit only a small amount of their energy along the particle path, until reaching a maximum penetration depth where the remaining energy is lost rapidly over a short distance, (within the clinical target) a phenomenon termed a “Bragg Peak”. Protons present almost no exit dose thereby minimising the total energy deposited in surrounding healthy tissues. Conversely, photon radiation consists of high-energy electromagnetic waves that penetrate tissue and deposit dose along the entire beam path [4]. Dose delivery is maximal just below the skin surface and continues to be exponentially attenuated along the entire treatment path until exiting the body [5]. While IMRT offers significant advantages over conventional 3D conformal radiotherapy in terms of dose to the clinical target, PBT demonstrates measurable attenuation in dose to non-target, healthy tissues outside the clinical target compared with IMRT. The relative biologic effectiveness (RBE) of protons is nearly equivalent to that of high-energy X-rays (RBE = 1.1) and so there is very little difference in tumour response per unit dose between protons and photons. Thus, any potential benefit of protons in terms of local tumour control can only result through physical dose escalation. PBT is an emerging alternative to IMRT and represents a step change in the ability to minimise dose to out-of-field healthy tissues—of critical importance in paediatric patients.

The inherent risk for second cancers in highly conformal treatment delivery techniques, like IMRT or proton therapy, arises in part from the dose deposited due to scattered radiation. For 6-MV IMRT, the scattered photon dose is primarily caused by leakage through the linear accelerator head and internal scatter within the patient. By comparison, passive scattered proton therapy produces secondary neutrons that form the primary source of out-of-field radiation. Furthermore, out-of-field risks are usually much lower than the in-field risks since most second malignancies are seen within the main irradiated area [6]. In a further technological advance, intensity modulated proton therapy (IMPT) is implemented by active scanning of a narrow beam spot. By means of energy modulation of the spots, the penetration depth in tissue can be adjusted, thus offering an additional degree of freedom. Spot-scanned proton therapy therefore exhibits even lower neutron contamination and enhanced healthy tissue sparing, because it requires no scattering, collimation or compensation [7]. IMPT was also found to reduce the estimated risk of secondary cancer induction and the use of smaller numbers of fields further increased this advantage [8]. There is currently much debate as to whether IMRT or protons should be used in the treatment of paediatric CNS tumours. This comes at a time when IMRT can be considered to have almost reached the limits of its technological evolution while in contrast, there has only, relatively recently, been widespread clinical use of PBT and available follow-up data are not yet sufficiently mature. In this review, we compare the differences in clinical outcomes of paediatric CNS patients treated with either protons or IMRT. In particular, tumours such as craniopharyngioma, medulloblastoma, low grade gliomas and ependymoma are examined.

## 2. Methods and Materials

### 2.1. Search Strategy and Selection Criteria

A literature search was performed for clinical studies reporting on the outcomes of paediatric patients with CNS tumours treated with protons or IMRT. The search terms used were protons, IMRT, CNS and paediatric. The titles and abstracts of articles were queried using search terms combined with Boolean operators “AND” and “OR” to produce more relevant results. The search was carried out in the following electronic databases: PubMed, ScienceDirect and HighWire in October 2014 and updated the following month. Reference lists of papers retrieved were also screened in order to provide additional potentially relevant papers not found in the main search. The following inclusion criteria were applied to the clinical studies in this review: (1) patients with CNS cancers; (2) paediatric patients only and (3) consider both IMRT and protons. In general, there was no limit placed on publication year or study design, but only papers published in English were considered.

### 2.2. Quality Assessment

A literature search was performed for clinical studies reporting on the outcomes of paediatric patients with CNS tumours treated with protons or IMRT. All relevant papers were assessed by both authors for the quality of the methods and for the results of key outcomes, which were identified and tabulated. One author (KSA) extracted data and this was independently checked by (NT) to ensure validity. Data was collated along the following criteria: study design, number of patients, age-range of patients, radiotherapy technique, target dose prescription and organ at risk constraints.

## 3. Results

### 3.1. Search Results

Of the three databases searched 1514 potential matches including duplicates were found. Of this total, 729 papers were from PubMed, 633 from ScienceDirect and 152 from HighWire. After an initial title screening, 189 potentially relevant papers were identified. Of these, 46 were excluded, 112 were retrieved as full papers and 31 as summaries only. After a second screening of both title and abstract applying the inclusion criteria outlined above, 10 eligible full papers were selected for this review [6,9,10,11,12,13,14,15,16,17]. All papers directly compared outcomes from protons with photons, five papers included medulloblastoma, four papers each included craniopharyngioma and low grade glioma and three papers included ependymoma.

### 3.2. Description of Selected Studies

Ten studies were selected of which two presented real patient outcomes (Table 1) and eight presented the results of dosimetric re-planning studies and modeling (Table 2). All 10 studies compared protons with IMRT for treating various paediatric CNS tumours. Of these 10 studies (*n* = 272), the largest clinical cohort included 120 patients [9] and the largest dosimetric re-planning cohort included 52 planning cases [11]. All studies directly compared proton with photon radiotherapy in paediatric patients or whole-body computational phantoms ranging in age from 0.8 to 18 years of age.

The earliest data included treatments from 1996, pre-dating the more widespread introduction of proton beam therapy. The majority of proton beam therapy data was found in the period 2004 to 2014. Proton beam treatment plans tended to have a minimum of three fields up to a maximum of six fields used in one study [16]. IMRT treatment plans by comparison, mostly used five or more fields up to a maximum of 11 fields. One study however, used only three or four fields to produce clinically acceptable IMRT treatment plans [10]. For diagnoses where focal radiotherapy is indicated, (craniopharyngioma, low grade gliomas and ependymoma) prescribed doses were mostly in the range 50 to 54 Gy. However, two related studies reported on a prescribed dose of 23.4 Gy for the craniospinal irradiation component of treatment for medulloblastoma [10,17].

### 3.3. Medulloblastoma

Five clinical studies were identified as reporting on paediatric medulloblastoma patients treated with protons or IMRT (Table 1). The single largest patient cohort included in our review was taken from a prospective study carried out by Yock *et al.* [9]. The Health-Related Quality of Life (HRQoL) was calculated for 57 paediatric brain tumor patients treated with PBT from 2004 to 2009 and for 62 paediatric brain tumour patients treated with photon therapy from 2001 to 2002. The age range was 2 to 18 years. The median follow-up was 2.9 years and there was no significant difference between the two cohorts in terms of age, gender, surgery type, diagnostic group or tumor location. This study considered medulloblastoma, ependymoma and low grade glioma diagnostic categories. In the medulloblastoma group, the proton cohort scored 9.8 post-treatment HRQoL points more than the photon cohort for total core score (*p* = 0.05). The proton cohort total core score (75.9) was 5 points less than the healthy population. When considered in the context of children with other chronic diseases such as diabetes (76.6), obesity (75.0) and asthma (68.8) or all cancers including leukemias (68.5), the PBT cohort compares favourably. By contrast, the photon cohort’s total core score was 65.4 and 15.5 points lower than the healthy controls (*p* < 0.001). The post-treatment HRQoL scores of the proton and photon cohorts were also compared directly. The proton cohort scored 10.5 points higher for the total core score [9].

**Table 1:** Description of Proton *versus* IMRT patient outcome studies on paediatric CNS patients. [Key: EPE—Ependymoma, LGG—Low Grade Gliomas, MED—Medulloblastoma].

Study	“*n*” of Patients	Age Range (Yr.)	Years	Radiotherapy Technique Protons IMRT		Diagnosis	Target Dose Px	Main Findings
Yock *et al.* [9]	120 (57/63)	2–18	(Protons) 2004–2009 (Photons) 2001–2002	various	various (not all IMRT)	MED EPE LGG	50–54 Gy	Health Related Quality of Life (HRQoL) for the proton cohort was 5 points less than the healthy population, whereas the photon cohort was 15.5 points lower.
Zhang *et al.* [10]	17	2–18	2007–2009	3–5 fields	3–4 fields	MED	23.4 Gy (RBE)/23.4 Gy	Proton CSI has the potential to reduce the risk of radiogenic 2nd cancers and cardiac mortality by up to 6× and 2nd cancer mortality by up to 3×.

**Table 2:** Description of Proton *versus* IMRT dosimetric re-planning comparisons on paediatric CNS patients. [Key: ASC—Astrocytoma, CRA—Craniopharyngioma, EPE—Ependymoma, LGG—Low Grade Gliomas, MED—Medulloblastoma, OGL—Optic Glioma].

Study	“*n*” of Patients	Age Range (Yr.)	Years	Radiotherapy Technique Protons IMRT		Diagnosis	Target Dose Px	Main Findings
Bishop *et al.* [11]	52	8.9 median	1996–2012	various	various	CRA	50.4 Gy (RBE)/50.4 Gy	PBT and IMRT produced equivalent outcomes related to survival and solid and cystic disease control.
Boehling *et al.* [12]	10	5–14	2007–2009	3 fields	5–7 fields	CRA	50.4 Gy (RBE)/50.4 Gy	Proton therapy resulted in significant sparing of normal tissues.
Brower *et al.* [13]	3	Not specified	2012	3 fields	9–11 fields	LGG	50 Gy	Proton therapy is an effective modality for reducing the dose deposition to non-target tissues.
Moteabbed *et al.* [14]	6	4–15	2013	3–4 fields	5–7 fields	MED EPE CRA ASC	50.4–54 Gy	Choosing proton therapy for paediatric patients with brain tumors is highly beneficial when considering second malignancies.
Paganetti *et al.* [15]	8	4–14	2012	3–4 fields	6–7 fields	OGL	52.2 Gy (RBE)/52.2 Gy	Proton therapy shows an overall advantage when estimating the risk for developing a second malignancy within the irradiated area.
Merchant *et al.* [16]	40	Paediatric	2008	various	various	MED EPE CRA OGL	54 Gy	A reduction in the mean dose from protons would have long-term clinical advantages for children with MED, CRA and OG.
Athar *et al.* [6]	6	0.75–14	2010	6 fields	6 fields	Cranial region	54 Gy	Protons can offer the advantage of a lower integral dose compared with IMRT.
Brodin *et al.* [17]	10	4–15	2007–2009	3 fields	2 Arc fields	MED	23.4 and 36 Gy	IMPT plans, including secondary neutron dose contribution, compared favourably to the photon techniques in terms of all radiobiological risk estimates.

The retrospective study conducted by Zhang *et al.* examined 17 paediatric medulloblastoma patients treated between 2007 and 2009 [10]. This study compared the risks of radiogenic second cancers and cardiac mortality for patients treated with passively scattered protons or field-in-field photon craniospinal irradiation. The Biological Effects of Ionization Radiation VII report and a linear model based on childhood cancer survivor data were used for risk predictions of second cancer and cardiac mortality respectively. The ratios of lifetime attributable risk (proton/photon) ranged from 0.10 to 0.22 for second cancer incidence and ranged from 0.20 to 0.53 for second cancer mortality. The ratio of relative risk for cardiac mortality ranged from 0.12 to 0.24. Proton therapy was found to confer significantly lower risks of cardiac mortality than photon therapy (*p* < 0.001) [10].

Moteabbed *et al.* performed a retrospective planning study involving six paediatric patients previously treated with passive scattered protons [14]. This study evaluated the risk of second cancer incidence in the vicinity of the primary radiation field for paediatric patients and compared passive scattering and pencil beam scanning proton therapy with IMRT. Dose distributions from this planning study were used to calculate the lifetime attributable risk for developing a second tumour in soft tissue and the skull. It was found that the lifetime attributable risk ranged between 0.01% and 2.8% for passive scattered and pencil beam scanned protons and between 0.04% and 4.9% for IMRT. The lifetime attributable risk for IMRT relative to passive scattered protons ranged from 1.3 to 4.6 for soft tissue and from 3.5 to 9.5 for the skull. Interestingly, the number of treatment fields used in proton therapy and IMRT were found to have minimal effect on the overall risk.

Another retrospective study by Merchant *et al.* included 40 paediatric patients, 10 each with optic pathway glioma, craniopharyngioma, ependymoma and medulloblastoma [16]. Dose volume data were collected for the whole brain, temporal lobes, cochlea and hypothalamus from each patient. The data were averaged and compared based on treatment modality using dose-cognitive effects models and outcomes were followed up over 5 years. Relatively small critical normal tissue volumes such as the cochlea and hypothalamus can be spared from radiation exposure when not adjacent to the primary target. Larger normal tissue volumes such as the supratentorial brain or temporal lobes receive less of the low and intermediate doses. These differences resulted in clinically significant higher IQ scores for patients with medulloblastoma [16].

Brodin *et al.* compared the risk of radiation-induced adverse late effects in 10 paediatric patients from 2007 to 2009 with medulloblastoma treated with either inversely-optimized arc therapy, (in this case RapidArc^®^, (Varian Medical Systems, Palo Alto, CA, USA) or spot-scanned intensity-modulated proton therapy (IMPT) [17]. Treatment plans were retrospectively created generated for two prescription craniospinal irradiation (CSI) doses, 23.4 Gy and 36 Gy. The risks of all long-term complications were shown to be considerably lower for IMPT than for the photon technique. Paediatric medulloblastoma appears an appropriate indication for PBT in terms of reduction in late effects due to irradiation of structures outside the planning target volume (PTV). These planning studies suggest a potential for reduction in second malignancy and cardiac mortality risks. This conclusion holds even for high values of neutron-influenced RBE. Superior quality of life measures have also been demonstrated, (in a prospective study). As the successful management of medulloblastoma includes surgery and chemotherapy as well as timely radiotherapy, it is important that the integrity of the whole clinical pathway is maintained to preserve high cure rates when considering PBT for medulloblastoma.

### 3.4. Craniopharyngioma

We considered four clinical studies reporting on paediatric craniopharyngioma patients treated with protons or IMRT (Table 1). The largest included a cohort of 52 (*n* = 21 for PBT and *n* = 31 for IMRT) paediatric patients treated between 1996 and 2012 and retrospectively compared by Bishop *et al.* [11]. Endpoints were overall survival, disease control, cyst dynamics and toxicity. At 59.6 months median follow-up, the 3-year outcomes were 96% overall survival, 95% for nodular failure-free survival and 76% for cystic failure-free survival. Neither overall survival nor disease control differed between treatment groups (overall survival *p* = 0.742, nodular *p* = 0.546, cyst *p* = 0.994). Immediately after therapy, 17 patients (33%) had cyst growth but more commonly in the IMRT group, (42% *vs.* 19% for PBT). Toxicity did not differ between both groups, (follow up was too short to demonstrate any reduction in late effects which may be anticipated from the use of PBT). Delaying radiation therapy for either treatment modality until recurrence may result in worse visual and endocrine function among paediatric patients with craniopharyngioma [11].

Boehling *et al.* retrospectively compared 10 paediatric patients aged 4 to 17 years old, with histologically diagnosed craniopharyngioma treated between 2007 and 2009 [12]. IMRT and IMPT treatment plans were generated and optimized with a prescribed dose of 50.4 Gy delivered in 28 fractions. PTV target coverage was adequate for both modalities with no significant differences for V_90%_, V_95%_ or V_100%_. Regions or normal brain outside the PTV, which were at risk for secondary malignancies, all had significant reductions in dose with the use of proton therapy. Intensity modulated proton therapy had the greatest effect on infratentorial brain, reducing integral dose by a factor of 2.1 compared with IMRT. This study did not account for the potential effect of secondary neutron dose from proton therapy. However, comparison between IMRT and protons, both active and passive scattering found that even taking into consideration the risks of neutron exposure, protons had lower risks of secondary malignancy than IMRT [12].

The Moteabbed *et al.* retrospective planning study involved six paediatric patients previously treated with passive scattered protons [14]. This study evaluated the risk of second cancer incidence in the vicinity of the primary radiation field for paediatric patients and compared passive scattering and pencil beam scanning proton therapy with IMRT. For craniopharyngioma patients, proton therapy was dosimetrically superior to IMRT especially at the lower dose region of the dose volume histogram curves. Approximately 1.5 to 4 times less volume of soft tissue and 5 to 6.5 times less skull was irradiated by protons relative to photons [14].

Ten patients with craniopharyngioma were included in the cohort of 40 analysed by Merchant *et al.* [16]. They reported that for relatively small critical normal tissue volumes that were anatomically separated from the PTV, such as the cochlea, the mean dose approached zero. For relatively large normal tissue volumes only partially intersected by the PTV and distinctly separate from the clinical target volume such as the infratentorial brain, or more fully subtended by the PTV, (temporal lobes, supratentorial brain, entire brain)—the high dose volume was minimally reduced, intermediate dose volume was moderately reduced and the low dose volume was substantially reduced. The dose to the hypothalamic-pituitary axis predicts endocrinopathy and for craniopharyngioma patients, doses to this region were found to be sufficiently large regardless of treatment modality to result in growth hormone deficiency [16]. These studies confirm the potential for proton beam therapy to reduce late effects of radiotherapy in craniopharyngioma, while maintaining tumour control rates comparable to IMRT.

### 3.5. Low Grade Glioma

We considered four clinical studies reporting on paediatric low grade glioma patients treated with protons or IMRT (Table 1). The large (*n* = 120) study conducted by Yock *et al.* also reported on the (Health Related Quality of Life) HRQoL outcomes of paediatric low grade glioma patients [9]. The post treatment HRQoL scores for the proton patients (*n* = 6) were consistently higher than the photon cohort (*n* = 12). In particular, it was found that for paediatric low grade glioma patients the HRQoL associated with protons was 86.7 of the total core score compared with 63.8 for the photon cohort. Protons also scored higher than photons in the sub-domains such as physical summary score, psychosocial summary score, emotional functioning, social functioning and school performance.

Brower *et al.* performed a retrospective treatment planning comparison of highly conformal radiation therapy for three paediatric biopsy-proven low grade glioma patients [13]. In all cases, target coverage goals were met. Although the majority of healthy tissue received considerably less dose with proton therapy, doses to most of the organs at risk, (including the cochlea) were well below acceptable institutional standards for both modalities. For paediatric low grade glioma patients, reducing the dose deposited to the surrounding uninvolved tissues may reduce late sequlae and proton beam therapy represents an effective modality for achieving this.

Eight optic pathway glioma (OPG) paediatric patient scenarios based on computational phantoms were included in the study by Paganetti *et al.* [15]. PBT plans used 3 fields while IMRT plans were optimized for six or seven fields and a prescribed dose of 52.2 Gy (RBE) applied to both modalities. Lifetime attributable risks for developing a second malignancy were calculated using a risk model considering cell kill, mutation and repopulation as well as inhomogeneous organ doses. For standard fractionation schemes, the lifetime attributable risk for developing a second malignancy from radiation therapy alone was found to be up to 2.7% for a 4 year old optic glioma patient treated with IMRT. The corresponding figure for protons was a factor of at least 2 (and up to 10) lower. This is mainly due to lower total energy deposited in the patient when using proton beams.

Merchant *et al.* compared models of dose characteristics and their relationship to cognitive function in a cohort of 40 paediatric patients, 10 of whom were diagnosed with OPG [16]. The OPG cases chosen for this study included patients with juvenile pilocytic astrocytoma and those with classically appearing optic pathway tumours that did not require histologic confirmation. They concluded that for relatively small critical normal tissue volumes within or immediately adjacent to the planning target volume, (optic chiasm, hypothalamus) the dose did not change. However, for small critical normal tissue volumes anatomically separate from the planning target volume, (cochlea) the dose was substantially reduced. For relatively large normal tissue volumes only partially intersected by the planning target volume, the high dose volume was moderately reduced and was less than 20% using protons [16].

### 3.6. Ependymoma

We considered three clinical studies reporting on paediatric ependymoma patients treated with protons or IMRT (Table 1). Yock *et al.* reported on the Health Related Quality of Life (HRQoL) outcomes of paediatric ependymoma patients treated with either protons or photons [9]. The Pediatric Quality of Life Inventory core score for the proton cohort (*n* = 15) was 77.1 and 59.2 for the photon cohort (*n* = 12) (*p* = 0.23). Protons also scored higher than photons for physical summary score (79.6/66.7), psychosocial summary score (75.8/55.0), emotional functioning (77.0/50.0), social functioning (82.7/57.9) and school functioning (67.7/56.7) for protons *versus* photons.

Moteabbed *et al.* determined the risk of radiation-induced second cancers in the high to medium dose region comparing passive and scanned proton therapy and IMRT in a cohort of six paediatric patients [14]. For a 4 year-old patient given a prescribed dose of 54 Gy, the mean dose to the uninvolved soft tissue using proton beam therapy was 12.1 Gy, compared with 20.9 Gy for 5-field IMRT. For the same patient the skull mean dose was 2.4 Gy and 13.5 Gy respectively for the same two modalities. The calculated absolute lifetime attributable risk for skull sarcoma was 0.06% for protons and 0.38% for 5-field IMRT. The lifetime attributable risks for soft tissue carcinoma were 1.08% and 1.91% respectively.

Ten paediatric patients diagnosed with ependymoma were among a cohort of 40 investigated by Merchant *et al.* [16]. They found that for relatively small critical normal tissue volumes that were anatomically separated from the PTV, (cochlea, chiasm, pituitary, hypothalamus) the dose was substantially reduced. The difference between protons and photons was such that the cochlea was entirely spared from high doses. The difference in the low dose volume was a factor of 3 in favour of protons. The dose difference for the chiasm, pituitary and hypothalamus was also reduced by a factor of 2 using protons thus demonstrating that protons have the potential to reduce hearing loss and endocrine effects.

## 4. Discussion

### 4.1. Clinical Studies and Dosimetric Re-Planning Comparisons

PBT has a theoretical advantage over photon radiotherapy for CNS tumours due to the ability of the former to deliver radiotherapy with steeper dose gradients to proximal critical structures than can be achieved with photons. Planning studies comparing conformal photon and PBT CNS radiotherapy techniques have found that PTV coverage is similar for both modalities but superior critical structure avoidance and lower total integral dose were much more apparent with PBT [18]. Our review has focused on 10 comparative proton *versus* IMRT studies with a total patient cohort of *n* = 272.

### 4.2. Additional Literature Reports

In addition to these, we appraised a further 13 studies (*n* = 96) which comprised a mixture of modeling studies and cohort studies with relatively short follow-up. No significant, concluded randomised clinical trials (RCT’s) were identified and these are unlikely to be possible in the future in paediatric patients due to unacceptability of randomisation between PBT and other photon based RT modalities. Patient cohorts in these smaller studies tend to be heterogeneous and often derived from a single centre [19,20,21,22,23,24,25,26,27,28,29,30]. A search for ongoing studies investigating the use of protons in paediatric patients with CNS tumours yielded seven results. The largest being a single centre study collecting data on normal tissue toxicity for proton therapy for paediatrics and will conclude in 2020 when the target cohort (*n* = 800) is met [31].There has been much debate as to whether protons should be used outside the framework of RCT’s. Critics of proton therapy highlight that the substantial investments required, (compared with photon therapy) are not supported by results from clinical trials [32].

### 4.3. Randomized Trials

According to Goozner, an Agency for Healthcare Research and Quality technical brief, published in 2009, identified 243 published articles based on clinical trials using proton beam therapy. Of these, 220 were single-arm studies and 185 were retrospective analyses. Just eight comparative, RCT’s involving PBT have taken place, and most of those compared different doses of protons, rather than comparing PBT with other modalities [33]. None of these included paediatric diagnoses. Large-scale comparisons between protons and photons for the treatment of paediatric CNS tumours would not meet the ethical basis upon which RCTs are conducted—namely *clinical equipoise*. An RCT should begin with a null hypothesis and there should be no compelling evidence that one treatment modality will be superior to existing treatments or indeed effective at all. This is not the case with protons where there are potential advantages relating to a superior dose distribution [34]. The Evaluation Subcommittee of ASTRO’s Emerging Technologies Committee report that, given the improvements in dose distribution and the well-established negative effects of dose in normal tissues in children, a randomised (PBT) trial may not be practically or ethically feasible [18].

### 4.4. Radiobiology of Protons

An emerging topic of discussion is the question of the validity of the widely accepted proton Relative Biological Effectiveness (RBE) relative to photons of 1.1 for the treatment of paediatric medulloblastoma [35,36]. It has been questioned whether the use of this generic RBE within tumours and normal tissues disregards the evidence that proton RBE varies with linear energy transfer (LET), physiological and biological factors. A broad analysis of RBE for cell survival conducted by Paganetti supports an average RBE for cell survival in the centre of a typical spread-out Bragg peak (SOBP) of approximately 1.15 for 2 Gy Fractions [37]. It was found that proton RBE increases with increasing LET and thus with depth in an SOBP. Values ranged from approximately 1.1 in the entrance region, to 1.15 in the centre, 1.35 at the distal edge and to 1.7 in the distal fall-off. However, data on RBE for endpoints other than clonogenic cell survival are too diverse to support use of a more specific proton RBE. Sethi *et al.* reported on failure patterns in 109 patients with medulloblastoma treated with PBT. They found no relationship between RT technique, proton end of range, LET or RBE in the 16 patients who exhibited a relapse during the median follow-up of approximately 40 months [38].

### 4.5. Radiation Dermatitis

Due to significant reduction in the integral dose to healthy tissues compared with photons, there is a general misconception that protons offer a reduction in skin toxicity. One early study of radiation dermatitis in single-field proton beam therapy found skin toxicity in 89% of cases (*n* = 49) treated between 1985 and 1987. However, the use of multiple proton beam angles and the spread out Bragg peak is able to mitigate the severity of skin reactions. Moskvin *et al.* retrospectively analysed the treatment records of 90 patients (median age 11.2 years) treated with PBT for brain and/or spine metastases from 2003 to 2011 and found that 77 (86%) had acute skin reactions. This included 86% of patients with ependymoma (*n* = 28) and 95% of patients with medulloblastoma (*n* = 21). The majority of patients, 87% experienced either Grade 1 or 2 skin toxicity. Among the youngest patients aged <5 years, 85% experienced Grade 1 toxicity. In contrast to megavoltage photon therapy, their results showed that the severity of acute skin reaction correlated with higher proton beam energies, independent of its correlation with the skin dose. The influence of energy on the severity of skin toxicity suggests the presence of radiobiological mechanisms specific to proton beam interaction with the epidermis and secondary particle generation. In general, paediatric patients have a long life expectancy and it is therefore important to examine more closely the skin reaction mechanism in proton therapy [39].

### 4.6. Neutron Scatter Contribution

Neutron scatter has also been cited as a potential obstacle to proton beam therapy (PBT) in paediatric CNS patients. During PBT, protons undergo nuclear reactions in tissue yielding secondary particles of different types. One of these, neutrons, may travel large distances before inducing a dose deposition. This poses a theoretical risk to non-target organs located outside the treatment volume which may receive unwanted secondary neutron doses. Secondary neutron doses from passive scattered protons received by healthy tissues of proton therapy patients were assessed using Monte Carlo calculations and computational phantoms. A maximum neutron absorbed dose of 16.5 mGy was found at the level of the salivary gland for a whole treatment delivering 54 Gy (RBE) to the tumour of a one-year-old patient with an intracranial tumour. Doses were also found to decrease with increasing distance from the treatment volume and with patient age [40]. Athar *et al.* used Monte Carlo modeling to quantify neutron equivalent doses and associated lifetime cancer incidence risks for head and neck and spinal proton therapy. They used computational whole-body, (gender-specific and age dependent) voxel phantoms and their results showed that the maximum neutron equivalent dose to organs near the proton field edge was found to be approximately 8 millisieverts per Gray. They further demonstrated that organ-specific neutron equivalent doses are age (stature) dependent [41]. Similar figures were calculated by Moteabbed *et al.* where they deduced that for proton therapy, the neutron generated risk for the entire brain is approximately 0.4% of the risk considering the primary beam only, (4 millisieverts per Gray) [14].

In terms of technology, there has been a trend among some manufacturers to produce a single-room compact PBT system. These are designed to offer more cost-effective PBT solutions with a smaller footprint. One such system is based on a superconducting synchro-cyclotron producing 230 MeV protons beams with a single compact gantry. This reduction in physical size would normally increase concern over the impact of neutron scatter. However, Monte Carlo modeling of the neutron scatter dose contribution to an anthropomorphic phantom, (adult sized) produced neutron doses in organs lying close to the primary field, (oesophagus and heart in this case) ranging from 4.3 to 7.1 millisieverts per treatment Gray and introduced no significant increase in second cancer risk [42].

### 4.7. Cost-Effectiveness of Protons

The cost-effectiveness of PBT *versus* the more readily accessible IMRT is another topic of debate. The fundamental decision facing countries without PBT is whether to adopt proton therapy now, with the risk of making the wrong clinico-financial decision, or wait for the collection of more follow-up information to reduce the existing uncertainty, with the risk of giving patients suboptimal treatment. Grutters *et al.* used “real options analysis” as a decision-making tool to answer this question for the Netherlands healthcare system [43]. The advantage of real options analysis is that it not only considers whether the benefits of clinical technology outweigh its costs but also considers the option to postpone adoption of the technology. Their analysis demonstrated that the net gain of “adopt and trial” was higher than that of “delay and trial” and was optimal for a patient sample size of 200. For the Dutch setting, adopting proton therapy and undertaking a trial was the preferred option. 

Our review has found that the most favourable characteristics of PBT are superior critical structure avoidance and lower total integral dose, especially in paediatric patients. These very young patients could still expect to survive for several decades resulting in an increase in quality-adjusted life years (QALYs). Vega *et al.* modeled the cost-effectiveness of proton therapy compared with photon therapy specifically in the management of paediatric medulloblastoma [44]. A cost-effective analysis was performed from the societal perspective using Monte Carlo simulation. A population of paediatric medulloblastoma survivors aged less than 18 years was studied. They had received treatment at age 5 years and were at risk of developing 10 adverse events, such as growth hormone deficiency, coronary artery disease, ototoxicity, secondary malignant neoplasm and death. Costing data included the cost of investment and the costs of diagnosis and management of adverse late effects. Their results demonstrated that proton therapy was associated with higher QALYs and lower costs and dominated photon therapy. In one-way sensitivity analyses, proton therapy remained the more attractive strategy, either dominating photon therapy or having a very low cost per QALY year gained. However, this study did not address the question of whether the cost-effectiveness of protons in paediatric medulloblastoma translated to other paediatric malignancies and did not justify the expense of more proton facilities.

## 5. Conclusions

The paediatric solid tumour population potentially has much to gain from more widespread use of proton beam therapy. Overall the data we have reviewed demonstrates the superiority of protons over photons for CNS tumours in children in terms of late neurocognitive, behavioural, vascular effects, health-related quality of life scores, endocrine abnormalities and second tumours. As clinical evidence accumulates and follow-up data matures, there is increasing interest amongst clinicians, patients and their families. The increase in availability of proton beam therapy, (including intensity modulated proton therapy) will no doubt lead to increasing numbers of published studies comparing protons with photons including highly conformal/IMRT. Proton beam therapy is not yet established as standard of care for paediatric CNS patients but is likely to become so in the future.
